# Effect of virtual reality educational program on critical thinking disposition among nursing students in Egypt: a quasi-experimental pretest–posttest design

**DOI:** 10.1186/s12912-025-03488-w

**Published:** 2025-07-07

**Authors:** Mona Ahmed Mohamed Gabr, Wafaa Fathi Sleem, Nehad Saad El-wkeel

**Affiliations:** 1 Nursing Administration, Faculty of Nursing, Mansoura University, Bani-Ebaid Hospital, Mansoura, Egypt; 2 Nursing Administration, Faculty of Nursing, Mansoura University, Mansoura, Egypt

**Keywords:** Critical thinking disposition, Nursing students, Virtual reality, Nursing education, Innovative learning

## Abstract

**Background:**

Education has continuously evolved to keep pace with advancements in information technology. Virtual reality (VR) has emerged as an innovative and effective learning approach that enhances student engagement, particularly in developing problem-solving and critical thinking skills. Since its introduction in the early 1980s, VR has gained increasing attention in nursing education due to its potential to improve learning outcomes and skill acquisition. However, there is a need for further research to explore its impact on critical thinking disposition among nursing students.

**Methods:**

This quasi-experimental pretest-posttest study was conducted with a convenience sample of 190 nursing students from Damietta University’s Faculty of Nursing. Participants were assessed on their critical thinking disposition and VR-related knowledge at three time points: before the intervention, immediately after, and two months post-intervention. Aim: The VR-based educational program aimed to improve students’ critical thinking disposition and assess their knowledge of virtual reality before, during, and after the program.

**Results:**

A statistically significant improvement in students’ mean scores for critical thinking disposition was observed across all evaluations (*p* < 0.001). The effect size (η²*p* ≥ 0.14) indicated a substantial impact, reinforcing the effectiveness of the VR-based educational program in fostering higher-order cognitive skills. Additionally, students demonstrated increased retention of VR-related knowledge when assessed immediately after the program and again after two months.

**Conclusions:**

This study highlights the transformative potential of VR as an educational tool in nursing. VR-based learning promotes active engagement, knowledge retention, and skill development in a safe and controlled environment. By integrating VR programs into nursing education, institutions can enhance students’ critical thinking, collaboration, and communication skills, ultimately preparing them for real-world clinical practice. Given these findings, educational institutions should consider incorporating VR technology to support the continuous development of essential nursing competencies.

**Trial registration:**

This study is formally registered on ClinicalTrials.gov (Identifier Code: NCT06622811; Registration Date: 01/10/2024).

## Introduction

Education is a fundamental pillar of a thriving society, continuously evolving to integrate new teaching methodologies that enhance learning experiences. In the modern, technology-driven era, virtual reality (VR) has emerged as a transformative educational tool, offering immersive, three-dimensional (3D) environments that foster student engagement and motivation in learning. Initially developed for gaming and entertainment, VR has now expanded into various fields, including healthcare education, where it serves as an innovative approach to bridging the gap between theoretical knowledge and practical application [1].

In nursing education, VR provides students with interactive simulations that replicate real-world clinical scenarios, helping them develop critical skills such as problem-solving, leadership, decision-making, patient safety, and disaster triage. These simulations create a safe and controlled environment where students can refine their skills without jeopardizing patient safety [2]. The term “virtual reality” was coined by Jaron Lanier and refers to computer-generated 3D environments that offer sensory inputs, allowing users to interact with objects as if they were physically present. Using various tools, such as VR headsets, gloves, mobile devices, and simulation software, students can experience a fully immersive learning environment. Studies indicate that VR-based learning enhances memory retention, interactive engagement, and clinical decision-making, positioning it as a valuable tool in modern nursing curricula [3].

Virtual Reality (VR) has evolved from its early applications in entertainment to become a transformative tool in various fields, including education. Initially developed for gaming and entertainment, VR technology has significantly advanced in recent years, with its integration into medical and nursing education providing immersive, hands-on learning experiences [4]. In nursing education, VR offers students the opportunity to engage in simulated clinical scenarios, helping them develop critical skills in a safe and controlled environment. Studies have shown that VR simulations improve clinical decision-making and patient care competencies among nursing students [5]. This study aims to explore the effectiveness of VR in nursing curricula, emphasizing its potential to enhance both theoretical knowledge and practical skills.”

In the context of nursing education, VR is defined as a computer-based 3D simulation that enables students to refine their skills in a realistic setting without posing risks to actual patients. VR simulations enhance memory retention, promote interactive learning, and improve students’ problem-solving abilities. There are two main categories of VR: immersive and non-immersive. Immersive VR provides a fully interactive experience using VR headsets and motion-sensing gloves, while non-immersive VR offers a partially interactive environment controlled by keyboards, mice, and touchscreens [5].

VR-based learning offers several benefits, such as enabling students to apply theoretical knowledge in practice, study at their own pace, and develop confidence in their skills. Additionally, VR eliminates the fear of making mistakes, allowing students to practice in a safe and controlled environment. As a result, nursing students using VR simulations are better prepared.

Critical thinking disposition refers to an individual’s mindset and approach to problem-solving and decision-making. Developing critical thinking skills is essential for nursing students, as it enhances their ability to analyze information, collaborate with peers, and make sound clinical judgments. Encouraging students to engage in discussions, problem-solving activities, and interactive learning experiences can strengthen their critical thinking abilities [6].

There are three types of critical thinking dispositions: intellectual, social, and inborn. Intellectual disposition is characterized by curiosity, open-mindedness, and willingness to explore new perspectives. Social disposition is influenced by education, coaching, and professional training, fostering ethical and responsible behavior. Inborn disposition is developed during childhood and shaped by early experiences. Critical thinking disposition, a key component of clinical competence, refers to an individual’s mindset and approach to problem-solving and decision-making. Developing strong critical thinking skills is essential for nursing students, as it enables them to analyze complex clinical situations, collaborate effectively, and make sound judgments. Previous research has identified three types of critical thinking dispositions: intellectual (curiosity and open-mindedness), social (influenced by education and professional training), and inborn (shaped by early experiences). Given the increasing demand for nurses with advanced critical thinking abilities, innovative educational strategies such as VR must be explored to assess their impact on students’ cognitive development [7].

A skilled critical thinker possesses various competencies, including defining problems and evaluating evidence, avoiding emotional reasoning and biases, considering multiple perspectives, asking insightful questions, anticipating consequences, and applying evidence-based reasoning in decision-making [8] Focus on VR aspects directly relevant to the study.

Clarify the link between the theoretical framework and objectives.

Support all claims with citations or evidence. Eliminate repetitive definitions and benefits of VR. This study aims to investigate the effectiveness of a VR-based educational program in enhancing critical thinking disposition among nursing students. By evaluating students’ critical thinking development before and after exposure to VR learning experiences, this research seeks to determine whether VR-based education fosters higher-order cognitive skills and contributes to improved clinical competence.

## Research hypothesis

This study hypothesizes that implementing a VR-based educational program will significantly enhance nursing students’ critical thinking disposition, providing them with the necessary tools to navigate complex clinical situations effectively.

### Theoretical framework

Several learning theories have shaped modern education, particularly in the latter half of the 20th century. One such theory is the Information Processing Learning Theory, a cognitivist approach that views the human mind as an information-processing system operating based on structured mechanisms for encoding, storing, and retrieving knowledge [9]. This model, originally developed by Richard Atkinson and Richard Shiffrin, conceptualizes learning as a sequence of cognitive transformations, where information moves through sensory memory, short-term memory, and long-term memory to facilitate knowledge retention and application [10]. In nursing education, critical thinking disposition is fundamental for effective clinical decision-making. The Information Processing Learning Theory aligns with this need, as it emphasizes cognitive engagement, structured problem-solving, and decision-making processes [11]. The theory suggests that active interaction with educational content strengthens mental strategies, leading to improved analytical reasoning and problem-solving skills. Integration of VR-Based Learning in the Theoretical Framework. Virtual Reality (VR) enhances cognitive development by immersing students in interactive, high-fidelity simulations, allowing them to actively engage with clinical scenarios [12]. VR-based learning aligns with Atkinson and Shiffrin’s model by supporting sensory engagement (perceiving visual and auditory stimuli), working memory activation (analyzing and responding to patient scenarios), and long-term retention (internalizing clinical knowledge and skills). Studies have demonstrated that VR simulations significantly enhance cognitive processing, knowledge retention, and higher-order thinking skills in nursing education [13]. By integrating VR within this theoretical framework, nursing students can: Encode information effectively through realistic, experience-based learning. Strengthen cognitive pathways by repeatedly engaging with dynamic patient cases. Improve problem-solving abilities by making evidence-based clinical judgments in real-time scenarios. Thus, VR-based learning serves as an experiential cognitive tool that reinforces the core principles of the Information Processing Learning Theory, enabling students to apply critical thinking skills in complex, real-world healthcare settings.

## Methods

This study utilized a quasi-experimental pretest-post test design, incorporating six student groups. The sample included two groups of 32 students each and four additional groups totaling 32 students, resulting in a convenience sample. Participants were selected based on availability and willingness to participate, which is a practical approach given time constraints and accessibility considerations. Sample Size and Participants. The total student population was 403, and the sample size of 190 students was calculated using Steven Thompson’s equation to ensure statistical adequacy. “Additionally, 19 students were included in a pilot study to validate the research instruments before implementing the main intervention. These students were excluded from the main study sample and were not included in the statistical analysis. Inclusion and Exclusion Criteria: Participants were required to be actively enrolled in the second semester of the 2022–2023 academic year at Damietta University’s Faculty of Nursing. The inclusion criteria required students to have no prior exposure to VR-based educational programs, ensuring that the study assessed first-time interactions with this technology. Only regular nursing students (who are officially enrolled and attending classes) were considered eligible. Exclusion criteria included: 1. Students with prior experience in virtual reality-based learning or those who had previously completed similar training modules to maintain a homogenous baseline.2. Students with visual impairments, epilepsy, or medical conditions that could hinder their engagement with VR technology. 3. Students who were unwilling to participate or could not commit to the follow-up assessments. While convenience sampling facilitates recruitment, it may limit external validity since the sample was not randomly selected. Consequently, the findings may not be fully generalizable to the broader population of nursing students or healthcare professionals. Future studies could enhance external validity by employing random sampling techniques or including a more diverse sample to improve representativeness. Data Collection and Assessment. Participants completed assessments on critical thinking disposition and VR knowledge at three points: Pre-intervention (Baseline Measurement). Immediately post-intervention. Two Months Follow-Up. The data were collected using a structured questionnaire, administered via a secure online survey platform and in paper-based format for students without internet access. The collected data were anonymized and securely stored for six months in a password-protected database, ensuring confidentiality and compliance with ethical standards.

Follow-Up Period: A two-month follow-up was implemented to evaluate the short-term retention of knowledge and critical thinking skills post-intervention. This timeframe allowed for an immediate assessment of the intervention’s effectiveness while minimizing disruptions to students’ academic schedules. Although this short-term follow-up provides valuable insights, future research could investigate long-term retention (e.g., six months or one year) to assess the sustainability of learning outcomes and further strengthen the external validity of the study’s conclusions.

The study protocol has been formally registered on ClinicalTrials.gov (Identifier Code: NCT06622811; Registration Date: 01/10/2024).

### Participants

The study included nursing students enrolled in the second semester of the 2022–2023 academic year at Damietta University’s Faculty of Nursing. The total student population was 403, and the sample size of 190 students was calculated using Steven Thompson’s equation. Additionally, 19 students were included in a pilot study to validate research instruments [10].

$$n=\frac{N\:xP\left(1-P\right)}{\left(N-1\right)x\left(d^{2}/z^{2}\right)^{+}P\left(1-P\right)}$$

#### Study site

Damietta University Faculty of Nursing in Egypt.

The study was conducted at the Faculty of Nursing, Damietta University, which was established in 2018. The faculty offers a four-year nursing degree program, followed by a one-year internship training. The institution comprises eight academic nursing departments, including: Nursing Administration, Community Health Nursing, Critical Care Nursing, Psychiatric and Mental Health Nursing, Medical-Surgical Nursing, Maternity and Gynecological Nursing, Pediatric Nursing. The faculty operates across two main buildings: The Administrative and Academic Building Houses the library, administrative offices, and faculty departments. Includes offices for the dean and vice deans (Postgraduate Studies, Education & Student Affairs, Environment & Community Service). Contains lecture halls and seminar rooms for theoretical instruction. The Clinical and Laboratory Building Equipped with eight specialized nursing skills labs for hands-on training. Provides simulation-based learning environments for practical nursing procedures. The faculty accommodates approximately 1,100 nursing students, ensuring comprehensive theoretical and practical education.

### Outcomes and instruments

**The Students’ Knowledge about Virtual Reality (VR) Questionnaire** was developed by the researcher based on an extensive literature review, referencing works by Pluye (2009) [11], Andersson & Fejzi (2019) [12], ALFalah (2020) [13], Dangle & Maegdefrau (2020) [14], Landers et al. (2021) [15], Dulina et al. (2022) [16], and Ikhsan et al. (2023) [17]. This 51-item questionnaire assesses students’ knowledge across multiple domains: VR’s definition, history, and technology (8 questions); VR tools and benefits (7 questions); drawbacks and limitations (5 questions); VR’s application in nursing education (7 questions); VR usage examples (3 questions); the importance of VR for nursing students (6 questions); VR applications in healthcare (4 questions); barriers to VR adoption (5 questions); and VR’s impact on patient care and education (6 questions). The questionnaire includes True/False (31 items) and Multiple-choice (20 items) formats. Correct answers receive 1 point, while incorrect answers receive 0 points. The total score is calculated by summing all correct responses, with a maximum possible score of 51 points. The results are categorized as Unsatisfactory (< 60%), meaning a score below 30.6 points, and Satisfactory (≥ 60%), meaning a score of 30.6 points or higher [17].

**To assess nursing students’ critical thinking disposition**, the researcher used an adapted version of Boonsathirakul & Kerdsomboon (2021) [18], based on Facione (2000) [19]. The scale consists of 24 items covering seven dimensions: Truth-seeking (3 items), Open-mindedness (4 items), Analytical skills (3 items), Systematicity (4 items), Critical thinking self-confidence (4 items), Inquisitiveness (3 items), and Cognitive maturity (3 items). It employs a six-point Likert scale ranging from Strongly Disagree (1) to Strongly Agree (6). The total score is calculated by summing all responses across the 24 items, with a maximum possible score of 144 points. The results are categorized as Satisfactory (≥ 75%), meaning a score of 108 points or higher, and Unsatisfactory (< 75%), meaning a score below 108 points. This tool evaluates students’ cognitive engagement and reasoning abilities, particularly in the context of VR-based learning.

### Sociodemographic questionnaire

The questionnaire also collected demographic data, specifically: Gender (Male/Female).Age (in years). This information provided insights into the participants’ characteristics and allowed for an analysis of how demographic factors may influence VR knowledge and critical thinking disposition.

### Validity and reliability

To ensure content validity, the data collection instruments were evaluated by five expert professors from the Nursing Administration Department at Mansoura University. Based on their feedback, modifications were made to improve clarity, relevance, and appropriateness of the items. Reliability Assessment: The reliability of the instruments was tested using the test-retest method and measured by Cronbach’s alpha coefficient. The obtained reliability scores were VR Knowledge Test: 0.887 (indicating high reliability). Critical Thinking Disposition Questionnaire: 0.937 (indicating excellent reliability). These scores confirm that the questionnaires demonstrated strong internal consistency, ensuring that the results were reliable and reproducible.

### Data collection

The educational program covered a comprehensive range of topics, including Definition of Virtual Reality (VR), Advantages and Disadvantages of VR, Types of VR, Examples of VR Applications, VR Equipment and Software, Importance of VR for Nursing Students, Practical Applications of VR in Nursing, Barriers and Challenges to VR Implementation, Impact of VR on Patient Care and Nursing Education. The fieldwork was conducted from early February 2023 to the end of July 2023. Nursing students’ VR knowledge and critical thinking disposition were assessed at three time points: (1) Pretest – Before the program’s implementation. (2) Immediate Posttest – Right after completing the educational sessions. (3) Follow-up Assessment – Two months after the program’s completion. The California Critical Thinking Disposition Inventory (CCTDI) was also administered at these three stages to evaluate changes in students’ critical thinking disposition over time.

### The intervention

The VR-based educational program was developed and implemented at Damietta University’s Faculty of Nursing with its structure refined using preliminary data and assessment outcomes. The program involved 190 nursing students divided into six groups, four groups of 32 students each and two groups of 31 students. Sessions were held from 9:00 AM to 1:00 PM, each lasting two hours. The program employed a variety of teaching methods including interactive lectures, group discussions brainstorming sessions small-group work role-playing activities and audiovisual and simulation-based learning. Each session followed a structured format to maximize student engagement 1. Review of previously covered content 2. Delivery of new learning material 3. Practical application through hands-on activities 4. Summary and Q&A session. The VR sessions were conducted using Oculus Rift VR headsets which provide an immersive 3D environment for students. The program utilized Sim Nurse software specifically designed to simulate realistic clinical scenarios in nursing. The software offered interactive scenarios where students interacted with virtual patients performing patient assessments diagnoses and clinical decision-making in a simulated setting. The program employed audio-visual simulation providing dynamic and sensory feedback to enhance the students’ theoretical and practical understanding.

Each VR session lasted 60 min with dedicated time for practical activities and direct interaction with the technology ensuring students fully engaged with the VR content. Real-time feedback was provided through the system allowing students to receive immediate assessments of their performance which promoted critical thinking and clinical decision-making in the simulated environment. The sessions were conducted individually ensuring personalized interaction with the VR content. After completing the VR-based educational sessions students completed post-test assessments to measure their immediate knowledge retention and critical thinking disposition. Two months later a follow-up assessment was conducted to evaluate long-term retention of learning and development in critical thinking. The two-month follow-up period was chosen to assess retention of knowledge while minimizing disruption to students’ academic schedules.

### Ethical considerations

The implementation of the VR educational program and data collection process were formally approved through an official letter from the Faculty of Nursing at Mansoura University to the Faculty of Nursing at Damietta University. Institutional permission was obtained using the appropriate administrative channels. Prior to participation, all students received a clear explanation of the study’s purpose, objectives, and procedures. Ethical approval was granted by the Scientific Research Ethics Committee, Faculty of Nursing, Mansoura University. All procedures in this study were conducted in accordance with applicable laws and ethical guidelines. Ethical approval was obtained from the Research Ethics Committee, Faculty of Nursing, Mansoura University (Reference No. P 309). This study was done in accordance with the Declaration of Helsinki.

Informed consent and Confidentiality Each participant provided written informed consent before any data was collected. The study’s purpose was explained in simple and accessible language to ensure full comprehension. All collected data was strictly confidential and used solely for research purposes. Participants were assured of their right to withdraw from the study at any time, without providing any justification.

### Statistical analysis

The data was coded, entered, and analyzed using SPSS (Statistical Package for Social Sciences) version 25 (IBM Corporation, Armonk, NY, USA). Descriptive Statistics: Quantitative data were summarized using range, mean, and standard deviation (SD). Qualitative data were analyzed using frequencies and percentages to describe categorical variables. Inferential Statistics: The following statistical tests were applied based on the data type: (1) Chi-Square Test (χ²): Used for categorical data to compare frequencies and proportions between two or more groups. (2) Mann-Whitney Test (Z-value): Applied to non-parametric independent samples to compare the means of two groups. (3) Kruskal-Wallis Test (H-value): Used to compare the means of more than two independent groups when data did not follow a normal distribution. (4) Friedman Test (χ²-value): Employed to analyze repeated measures (pretest, posttest, and two-month follow-up) for non-parametric data. (5) Pearson’s Correlation Coefficient (r): Used to assess the relationship between VR knowledge and critical thinking disposition. All statistical tests were conducted with a significance level of *p* < 0.05, indicating that findings were considered statistically significant if the p-value was below this threshold.

## Results

Table 1 and 2 presents the demographic characteristics of the nursing students at Damietta University included in the study. The results indicate that the majority of participants were female (54.7%), while male students comprised 45.3%. The mean age of participants was 20.96 ± 0.74 years, with most students aged between 20 and 21 years. These demographic details provide a comprehensive understanding of the study sample and help assess the influence of these variables on educational outcomes.

**Table 1:** Virtual reality program

	Out line	Media	Methods of teaching	Methodsof evaluation
1	Introduction and feedback	Data show	Brainstorming Q&A Discussion	Question feedback
2	Definition of virtual reality	Data shows, animation, PowerPoint, videos	Interactive lecture	Posttest Question feedback
3	History of virtual reality	Data shows, animation, power point, videos	Lecture & discussion	Posttest Question feedback
4	Importance of virtual reality	Data show, power point, videos, simulation	Case based learning	Posttest Question Feedback
5	Tools used in virtual reality	Data Show, figures	Group discussion, power point	Question feedback
6	virtual reality programs	Data show, PowerPoint, videos	Interactive lecture	Posttest Question feedback
7	Types of virtual reality	Data show, PowerPoint, videos	Case studies & examples	Posttest Question feedback
8	Examples of virtual reality	Data show, PowerPoint, videos	Brainstorming, Interactive activities	Question feedback quiz
9	Applications of virtual reality in nursing education	Data show, power point, videos	Problem Based learning	Quiz
10	Advantages of virtual reality	Data show, Power point, videos	Interactive discussion	Posttest Question Feedback
11	Disadvantages of virtual reality	Data show, PowerPoint, videos, simulation	Case based discussion	Posttest Question Feedback
12	How virtual reality transforming nursing education	Data show, group discussion, power point	Reflective learning	Assignment
13	Challenges in implementing virtual reality	Data show, power point, videos	Group discussion, problem solving	Posttest Question Feedback
14	Barriers to virtual reality implementation	Data show, power point, videos	Case studies, problem solving	Posttest Question feedback + case study

**Table 2:** Personal characteristics of the nursing students studied (*n* = 190)

Personal characteristics	The studied nursing students (*n* = 190)
	No(%)
**Gender**	
Female	104(54.7%)
Male	86(45.3%)
**Age years**	
20	56(29.5%)
21	86(45.3%)
22	48(25.3%)
Range Mean ± SD	20–22 20.96 ± 0.74

Data was presented as percentage and Mean ± SD

**Table:** 

**Outcome**			

**Fig. 1:**
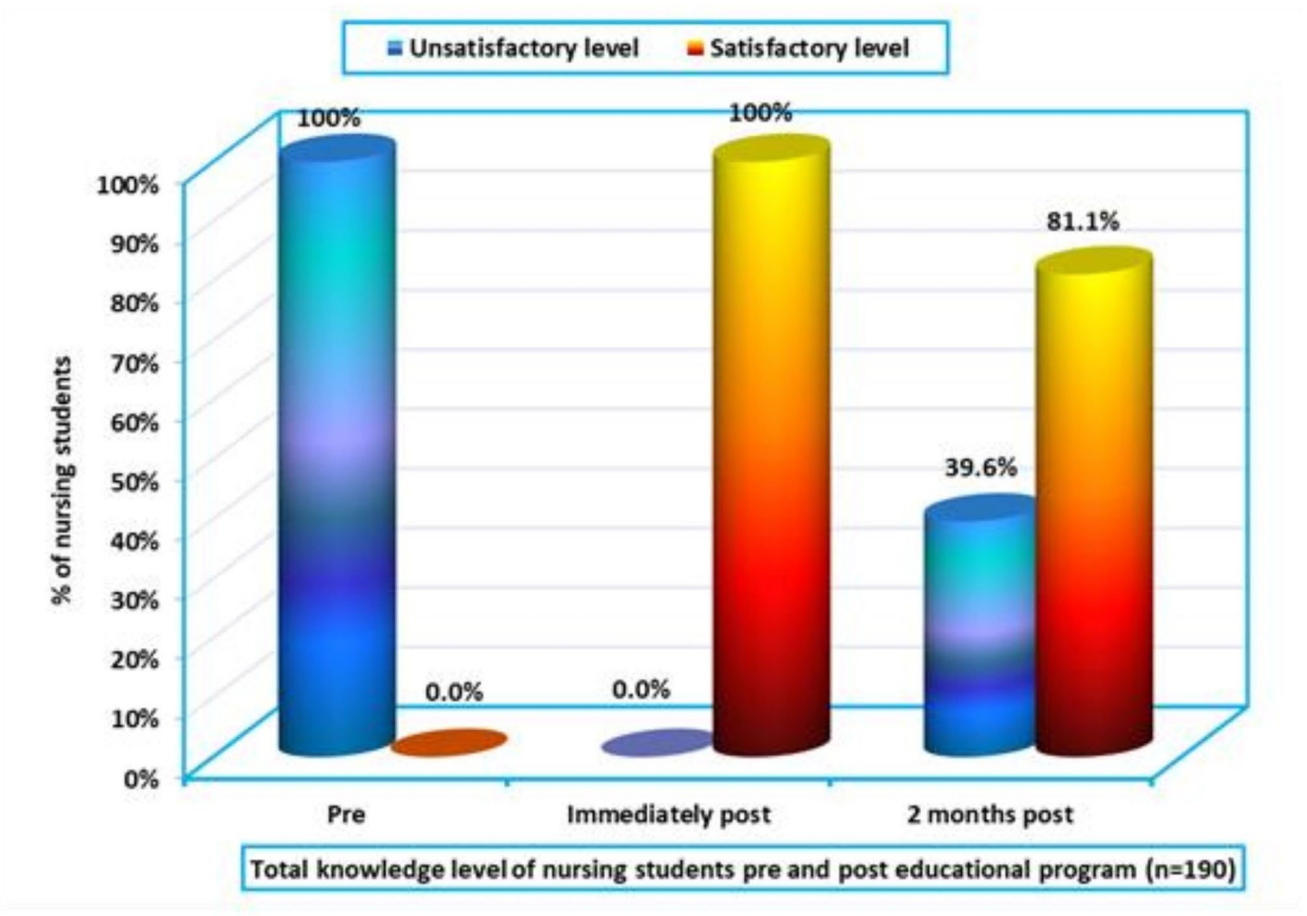
Total knowledge level of nursing students before and after the educational program (*n* = 190)

Figure 1 illustrates the students’ knowledge levels before and after the intervention. Prior to the VR-based educational program, 100% of students had an unsatisfactory knowledge level. However, in two months post-program, 81.1% of students demonstrated satisfactory knowledge, confirming a significant improvement in VR-related knowledge acquisition (Cohen’s d = 1.15, 95% CI = [0.95, 1.35]). This large effect size indicates a substantial impact of the educational program on students’ knowledge, highlighting its effectiveness.

**Fig. 2:**
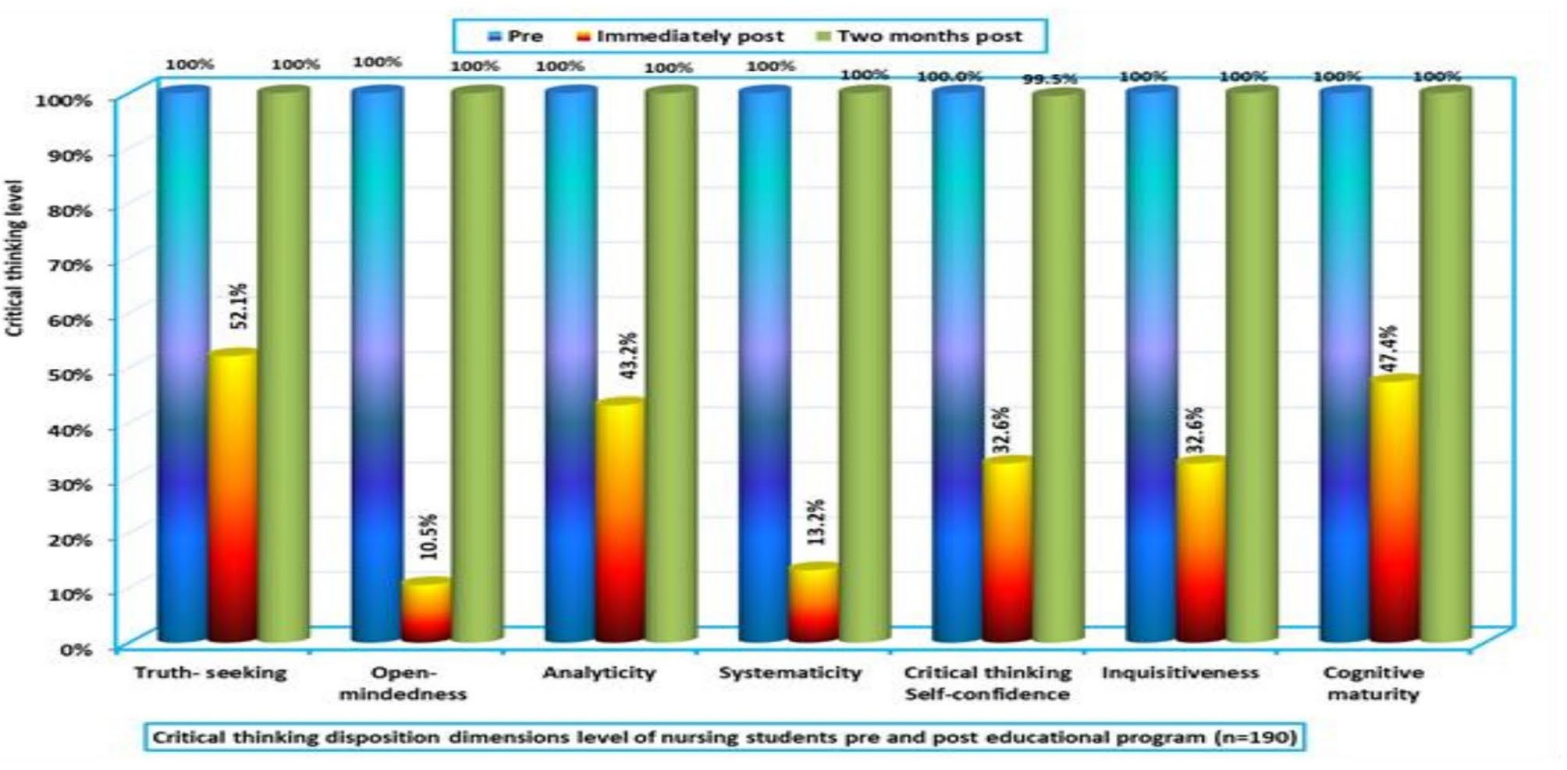
Levels of critical thinking disposition dimensions among nursing students before and after the educational program (*n* = 190)

Figure 2 presents the distribution of critical thinking disposition subdimensions before and after the educational program. The results indicate that all subdimensions showed a 100% improvement two months post-intervention. Additionally, during the immediate post-test phase, the majority of students (52.1%) exhibited truth-seeking behaviors, while only 10.5% demonstrated open-mindedness. These findings highlight the program’s influence in fostering critical thinking skills among nursing students.

**Fig. 3:**
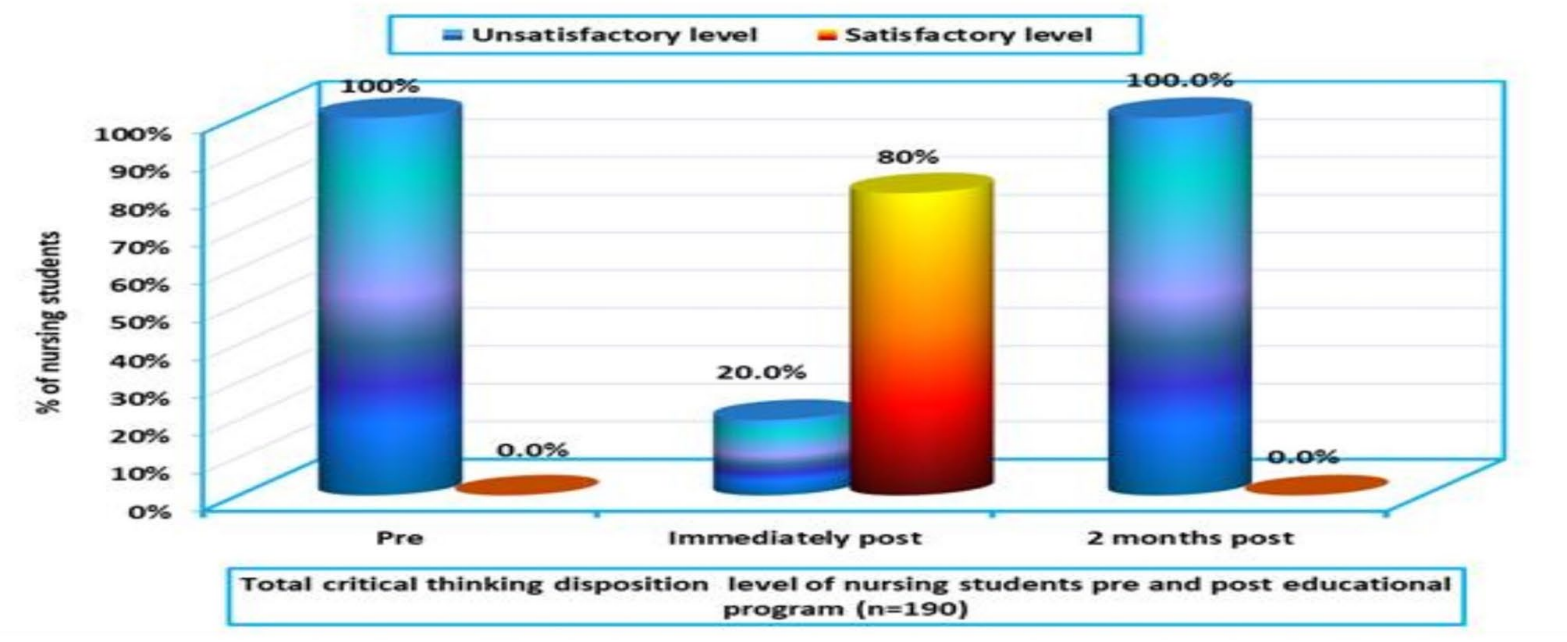
Overall critical thinking disposition levels of nursing students before and after the educational program (*n* = 190)

Figure 3 depicts the overall critical thinking disposition levels of nursing students before and after the educational intervention. Initially, 100% of students scored it in the “unsatisfactory” category. However, immediately after completing the program, 80% of students exhibited satisfactory critical thinking disposition, while only 20% remained in the unsatisfactory category. These findings reinforce the effectiveness of the VR-based learning approach in enhancing analytical and evaluative skills.

**Fig. 4:**
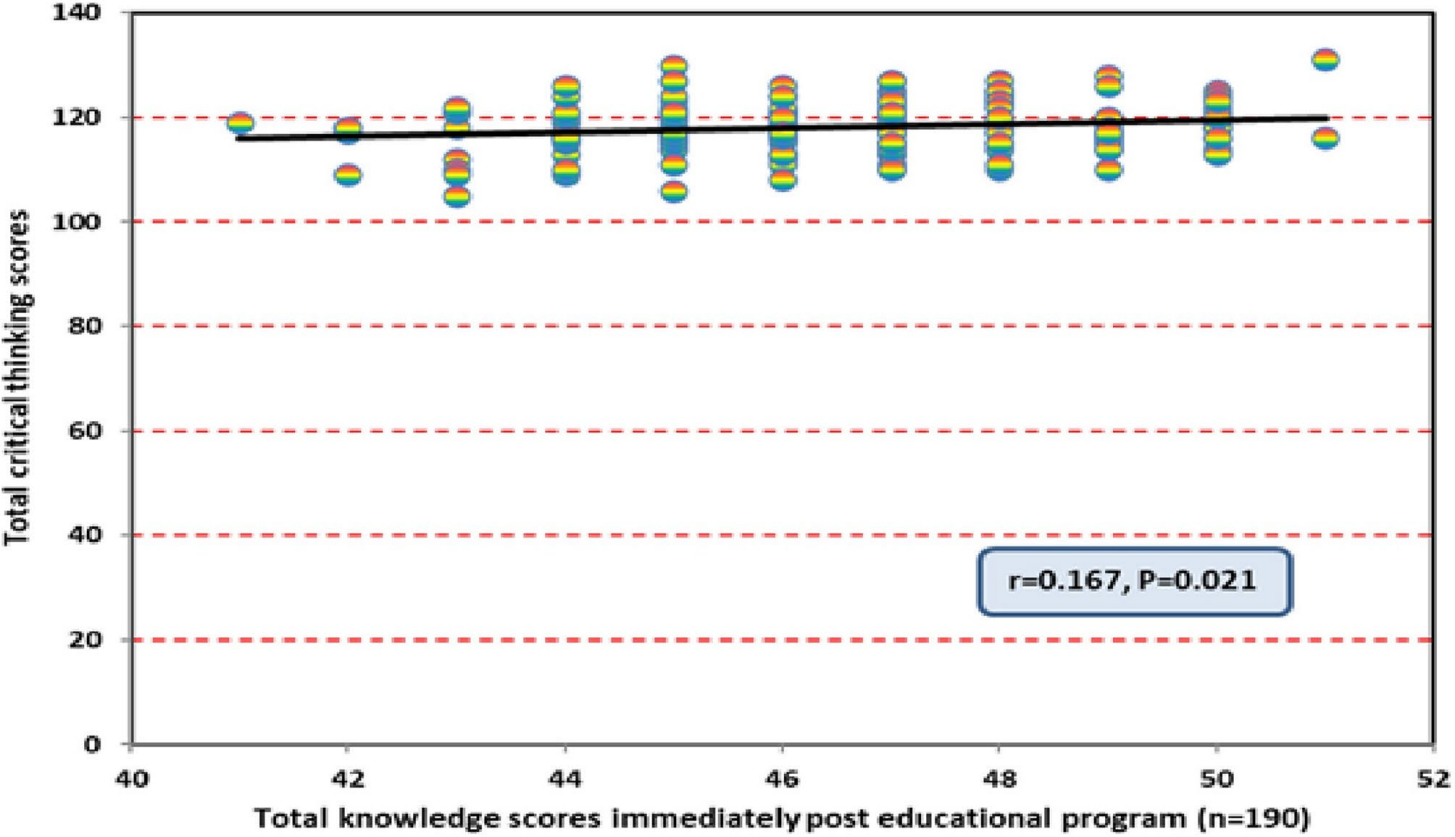
Correlation between total knowledge scores and total critical thinking disposition scores among nursing students immediately after the educational program (*n* = 190)

Figure 4 presents the correlation between total knowledge scores and total critical thinking disposition immediately after the VR-based educational program. The results reveal a statistically significant positive correlation, suggesting that students with higher VR knowledge also demonstrated enhanced critical thinking disposition. This relationship indicates that improved content understanding directly contributes to the development of higher-order thinking skills.

**Fig. 5:**
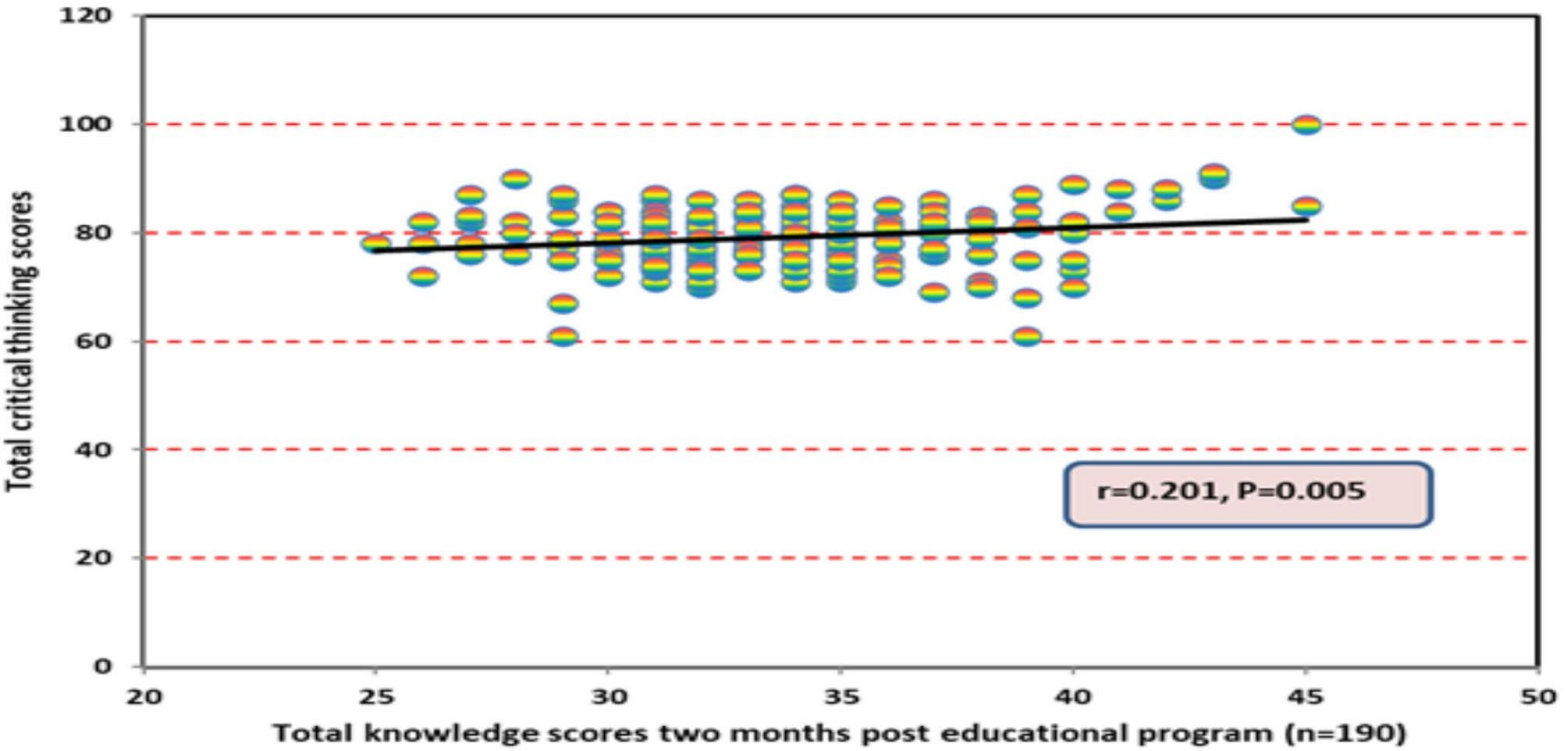
Correlation between total knowledge scores and total critical thinking disposition two months after the educational program

Figure 5 demonstrates the correlation between total knowledge scores and total critical thinking disposition two months after the educational program. A statistically significant positive correlation was observed, indicating that students retained both VR knowledge and improved critical thinking skills over time. These results underscore the long-term benefits of immersive learning environments in sustaining critical thinking abilities.

Table 3 reports the mean knowledge scores of nursing students and their relationship to personal characteristics before and after the educational program. The results showed that, after two months, total knowledge scores were significantly correlated with students’ gender and personal characteristics. However, no statistically significant relationship was found between personal characteristics and knowledge levels before or immediately after the program. These findings suggest that while gender and other personal traits may influence knowledge retention in the long run, the initial learning outcomes are primarily driven by the educational intervention itself.

**Table 3:** Total knowledge mean score of the studied nursing students and thier relation to personal characteristics(*n* = 190)

Personal characteristics		No.	Knowledge total score of the studied nursing students pre and post educational program		
			Pre	Immediately post	2 months post
			Mean ± SD	Mean ± SD	Mean ± SD
Gender	Female	104	6.55 ± 2.95	46.79 ± 1.75	33.12 ± 3.82
	Male	86	6.87 ± 3.12	46.23 ± 2.25	34.38 ± 3.86
Z value			0.988	1.879	2.309
P value			0.323	0.060	0.021*
Age years					
20		56	6.55 ± 3.13	46.5 ± 2.15	33.03 ± 3.38
21		86	6.58 ± 0.63	46.55 ± 1.95	34.46 ± 3.93
22		48	7.06 ± 3.57	46.56 ± 1.97	33.08 ± 4.14
x ^2^ value			0.864	0.132	4.795
P value			0.388	0.936	0.091

Data was presented as Mean ± SD

## Discussion

This study aimed to evaluate the effectiveness of a virtual reality (VR) educational program in enhancing the critical thinking disposition of nursing students. The results showed a significant improvement in students’ knowledge levels and critical thinking disposition after the implementation of the VR program. However, several factors should be considered when interpreting these findings. The study found that more than half of the participants were female. This can be explained by the fact that females in Egypt tend to enroll more frequently in educational and training programs compared to males. Additionally, societal perceptions play a significant role; nursing is traditionally seen as a predominantly female profession in Egypt, which may discourage male students from pursuing a nursing career due to long, irregular working hours and night shifts. Moreover, males may prefer other professions offering better financial incentives and social prestige. Historically, nursing education in Egypt was initially exclusive to females, but due to the increasing demand for nurses of both genders, nursing faculties later opened admissions to male students as well. It is important to consider the Hawthorne Effect in this study, which refers to the potential impact of students knowing they are part of an experimental study on their performance. In this study, it is possible that students showed improvement in their performance due to the awareness that they were part of a research project. This awareness may have encouraged them to focus more on the learning activities and improve their performance, which could partly explain the observed improvements in knowledge levels and critical thinking disposition. Although the study design attempted to control this, future research could benefit from using a control group that is unaware of being part of an experimental study to examine the effects of VR more precisely.

The study also found that less than half of the students were 21 years old. This result can be explained by the fact that the study sample consisted of fourth-year students, whose ages typically range from 20 to 22 years. Most students at this institution follow a regular academic progression, which aligns with this age range. These findings are consistent with those of Alrashidi et al. and Mahmoud & El-Saadany [20], who found that approximately half of the nursing students in their studies were between the ages of 20 and 22. However, the results differ from those of Li, D [21], who reported that a minority of nursing students were aged between 20 and 24 years. This discrepancy may be due to differences in study conditions, sample sizes, and cultural factors affecting enrollment in nursing education across regions.

The study found that after two months of implementing the VR-based educational program, most nursing students demonstrated satisfactory knowledge levels, with a statistically significant improvement compared to their pre-program knowledge levels. This improvement may be attributed to the fact that virtual reality is a relatively new teaching method, and students had no prior exposure to this technology before the intervention. Moreover, the positive impact of the VR program could be linked to the fact that traditional face-to-face learning methods often lead to student distraction. Knowledge acquired through conventional teaching approaches tends to fade over time, whereas VR-based simulators provide interactive and immersive experiences, allowing students to engage more effectively with the learning material. This interaction enhances long-term memory retention and enables students to form deeper cognitive connections with the subject matter. These findings align with those of Kazu & Kuvvetli [22], who reported a significant improvement in VR knowledge levels before and after an educational program, and Al-Ansi & Jaboob [23], who found similar improvements in students’ understanding of VR concepts.

The findings of the present study showed that the majority of participants initially exhibited a low level of critical thinking disposition. However, after two months of VR-based education, significant improvements were observed across all dimensions of critical thinking. This enhancement may be due to the novelty of VR as a teaching method, as students had no prior exposure to VR technologies. Furthermore, VR technology supports students in organizing, analyzing, and synthesizing information, while also helping them link theoretical knowledge to healthcare concepts. Unlike traditional passive learning methods, VR-based education promotes active learning, allowing students to engage more dynamically with the material. This engagement fosters a sense of accomplishment and confidence in learning, which plays a crucial role in enhancing critical thinking skills Söderström, Stensrud, & Siqveland [24]. These results are consistent with Baek & Lee [25], who found that nursing students demonstrated high levels of critical thinking and performance after participating in a VR-based educational program Chiange [26], Ikhsan, Sugiyarto, & Astuti [16], who showed that virtual laboratories enhance students’ learning outcomes. Additionally, Aminu, Ochanya, & Omilani [27] highlighted the significant impact of VR-integrated hybrid learning on students’ critical thinking abilities.

The results of this study revealed a statistically significant positive correlation between total knowledge scores and critical thinking disposition two months after the educational program. This correlation may be attributed to the fact that VR actively engages students, transforming them from passive recipients of information to active learners. VR enhances students’ sense of accomplishment and confidence, which plays a key role in developing critical thinking skills beyond what is typically achieved through traditional learning methods. Additionally, VR allows students to visualize complex and abstract concepts in a three-dimensional space, which improves problem-solving abilities and cognitive processing. This result aligns with Nemati-Vakilabad et al. [28] al., who found a positive correlation between total knowledge scores and critical thinking abilities in nursing students.

This study found a statistically significant correlation between students’ gender and their knowledge levels two months after the program. However, no statistically significant correlation was observed between students’ personal characteristics (such as age or social status) and their knowledge levels before or immediately after the program. These findings are consistent with Chen et al. [29], who found no statistically significant relationship between personal characteristics and pre- or immediate post-program knowledge levels. Similarly, Salam, Abdallah, & Abd El-Hay [30] reported no significant relationship between total knsaaowledge scores and demographic characteristics, except for gender. However, these results contrast with those of Plotzky et al. [24], who found a statistically significant relationship between students’ age and their knowledge of VR. This discrepancy may be due to differences in study populations, sample sizes, and the specific VR training content used in various educational settings.

This study contributes to the growing body of literature on the effectiveness of VR in nursing education, particularly in fostering critical thinking skills. While the results are promising, it is important for future studies to consider the Hawthorne Effect and control for other potential confounding variables, such as socioeconomic status and prior technology exposure. Future research could also explore the long-term impact of VR-based learning on other skills, such as clinical decision-making, and investigate the effectiveness of VR in more immersive and controlled environments. Moreover, studies could benefit from larger and more diverse sample sizes to generalize findings across various educational settings.

## Conclusions

The findings of this study provide strong evidence that virtual reality (VR) education can significantly enhance nursing students’ critical thinking disposition. Notably, improvements were observed not only in overall test scores but also in key cognitive domains, with sustained positive effects even two months after the intervention. These results confirm the effectiveness and feasibility of integrating VR technology into nursing education as a tool to cultivate critical thinking skills and encourage meaningful cognitive development among nursing students. The study underscores the potential of VR-based learning to promote engagement, enhance problem-solving abilities, and support informed clinical decision-making in nursing practice.

### Implications of the study

To keep pace with advancements in educational technology, nursing curricula and instructional strategies should be updated to integrate VR-based learning. This approach enables students to: Develop essential clinical skills in a safe and controlled environment. Enhance teamwork and communication in healthcare settings. Gain exposure to real-world challenges and risks, boosting confidence and preparedness for clinical practice. Furthermore, VR fosters active engagement, allowing students to experience realistic patient interactions and complex decision-making scenarios without compromising patient safety. By incorporating modern technologies into nursing education, educators can effectively promote critical thinking disposition (CTD), an essential competency for both academic success and clinical excellence. Since critical thinking is fundamental in nursing practice, nursing educators should prioritize innovative teaching methods that cultivate higher-order thinking skills, ensuring that students graduate with the cognitive abilities necessary to provide high-quality patient care.

### Study limitations and recommendations

This study has several limitations that should be acknowledged. First, the use of a convenience sample from a single faculty limits the generalizability of the results to broader nursing student populations. Additionally, the study focused on short-term outcomes, and the long-term effects of VR-based educational programs on critical thinking disposition remain unexplored.

To overcome implementation challenges, institutions must ensure faculty are adequately trained, not only in the technical use of VR tools but also in designing pedagogical strategies that promote critical thinking. Financial constraints and infrastructure demands — such as the cost of VR equipment, maintenance, and the need for dedicated spaces — present further limitations to widespread adoption. Continuous evaluation and feedback from both students and educators are essential for refining VR programs and ensuring their alignment with learning objectives. Finally, while VR enhances learning through immersive experiences, it should complement rather than replace traditional teaching methods to provide a comprehensive nursing education.

Future research should examine the long-term impact of VR on clinical decision-making, teamwork, and other core nursing competencies. Comparative studies across different institutions and student demographics are also recommended to identify the most effective VR applications in nursing education.

## Data Availability

The study’s quantitative datasets can be obtained from the corresponding author upon justifiable request.

## Abbreviations

VR: Virtual reality
CT: Critical thinking
CCTD: California Critical Thinking Disposition
